# CD73 Ectonucleotidase Restrains CD8+ T Cell Metabolic Fitness and Anti-tumoral Activity

**DOI:** 10.3389/fcell.2021.638037

**Published:** 2021-02-18

**Authors:** Pedro Briceño, Elizabeth Rivas-Yañez, Mariana V. Rosemblatt, Brian Parra-Tello, Paula Farías, Leonardo Vargas, Valeska Simon, César Cárdenas, Alvaro Lladser, Flavio Salazar-Onfray, Alvaro A. Elorza, Mario Rosemblatt, María Rosa Bono, Daniela Sauma

**Affiliations:** ^1^Departamento de Biologia, Facultad de Ciencias, Universidad de Chile, Santiago, Chile; ^2^Facultad de Medicina y Ciencia, Universidad San Sebastian, Santiago, Chile; ^3^Center for Integrative Biology, Universidad Mayor, Santiago, Chile; ^4^Fundacion Ciencia & Vida, Santiago, Chile; ^5^Disciplinary Program of Immunology, Institute of Biomedical Sciences, Faculty of Medicine, Universidad de Chile, Santiago, Chile; ^6^Millennium Institute on Immunology and Immunotherapy, Santiago, Chile; ^7^Institute of Biomedical Sciences, Faculty of Medicine and Faculty of Life Sciences, Universidad Andres Bello, Santiago, Chile

**Keywords:** CD73/NT5E, CD8 T cell, metabolism, cytotoxic, antitumor activity

## Abstract

CD39 and CD73 are ectoenzymes that dephosphorylate ATP into its metabolites; ADP, AMP, and adenosine, and thus are considered instrumental in the development of immunosuppressive microenvironments. We have previously shown that within the CD8+ T cell population, naïve and memory cells express the CD73 ectonucleotidase, while terminally differentiated effector cells are devoid of this enzyme. This evidence suggests that adenosine might exert an autocrine effect on CD8+ T cells during T cell differentiation. To study the possible role of CD73 and adenosine during this process, we compared the expression of the adenosinergic signaling components, the phenotype, and the functional properties between CD73-deficient and WT CD8+ T cells. Upon activation, we observed an upregulation of CD73 expression in CD8+ T cells along with an upregulation of the adenosine A2A receptor. Interestingly, when we differentiated CD8+ T cells to Tc1 cells *in vitro*, we observed that these cells produce adenosine and that CD73-deficient cells present a higher cytotoxic potential evidenced by an increase in IFN-γ, TNF-α, and granzyme B production. Moreover, CD73-deficient cells presented a increased glucose uptake and higher mitochondrial respiration, indicating that this ectonucleotidase restrict the mitochondrial capacity in CD8+ T cells. In agreement, when adoptively transferred, antigen-specific CD73-deficient CD8+ T cells were more effective in reducing the tumor burden in B16.OVA melanoma-bearing mice and presented lower levels of exhaustion markers than wild type cells. All these data suggest an autocrine effect of CD73-mediated adenosine production, limiting differentiation and cytotoxic T cells’ metabolic fitness.

## Introduction

Type 1 Cytotoxic CD8+ T (Tc1) cells are pivotal in the elimination of infected and transformed cells. The differentiation process that culminates in the generation of effector T CD8+ lymphocytes is not homogeneous, and during the immune response, there are T cell subsets with varying proliferative and cytotoxic capacities (Williams and Bevan, 2007; Gattinoni et al., 2012). Two populations can be distinguished following CD8+ T cell activation and clonal expansion: short-lived effector cells and memory effector cells (Kaech et al., 2003; Badovinac et al., 2004; Joshi et al., 2007). Short-lived CD8+ effector cells are characterized by a more significant commitment to the effector profile and a lower survival and self-renewal capacity than memory effector cells. Circulating memory cells are divided into two compartments: central memory lymphocytes (*T*_*CM*_) and effector memory T cells (*T*_*EM*_) (Sallusto et al., 1999; Sallusto et al., 2004). *T*_*CM*_ cells are characterized by a lower commitment to effector differentiation and the expression of receptors for migration to secondary lymphoid organs, such as CD62L and CCR7. *T*_*EM*_ cells, on the other side, present a higher commitment to an effector program and are capable of migrating and entering into non-lymphoid peripheral tissues. During T cell effector differentiation, the cells gradually upregulate transcription factors related to effector differentiation, such as T-bet, Blimp-1, and reduce transcription factors related to a less differentiated state such as TCF-7, Lef-1, Bcl-6, among others (Zhang and Bevan, 2011; Kaech and Cui, 2012).

The process of effector differentiation is characterized by a metabolic switch necessary to initiate the effector program and functions of cytotoxic T cells (van der Windt and Pearce, 2012; Cammann et al., 2016; Menk et al., 2018). Previous reports indicate that naive lymphocytes have a low energy demand, which they supply through oxidative phosphorylation, mainly through fatty acid oxidation (FAO) and small amounts of glucose to generate ATP (Jones and Thompson, 2007; MacIver et al., 2013). The acquisition of effector features by cytotoxic T cells results from a shift to a predominating glycolytic metabolism in detriment of FAO (Wang et al., 2011; van der Windt and Pearce, 2012; Hukelmann et al., 2016). This increase in energy demand results in increased glucose absorption, which contributes to the promotion of anabolic processes that enables cell growth, proliferation, and the production of effector molecules (Lunt and Vander Heiden, 2011; Chang et al., 2013; Pearce et al., 2013; Peng et al., 2016).

Following tissue damage, ATP is released to the extracellular space where it is rapidly hydrolyzed to adenosine by the tandem action of extracellular ectonucleotidases such as CD39 and CD73. The first step in ATP hydrolysis is catalyzed by CD39, which generates ADP and AMP (Robson et al., 2006). The second step involves the action of CD73, which hydrolyzes AMP into adenosine (Regateiro et al., 2013). It has been demonstrated that CD39 and CD73 are highly upregulated in the tumor microenvironment, which causes an increase in the intratumoral concentration of adenosine (reaching the micromolar range). Extracellular adenosine dampens the antitumor response by preventing the activation, proliferation, cytotoxicity, and cytokine production by activating A2A receptor on T cells (Huang et al., 1997; Deaglio et al., 2007; Linnemann et al., 2009; Ohta et al., 2009; Mastelic-Gavillet et al., 2019).

The expression of CD39 and CD73 ectonucleotidases was initially described in tumor cells, regulatory T cells (Tregs), and myeloid-derived suppressor cells (MDSCs), where they enhance their immunosuppressive function through the production of adenosine (Kobie et al., 2006; Borsellino et al., 2007; Deaglio et al., 2007; Li et al., 2017). However, human and murine CD8+ T cells also express these ectonucleotidases. In humans, naive CD8+ T cells express higher levels of CD73 than CD8+ memory T cells (Dianzani et al., 1993), and *in vitro* activation of PBMC has been reported to induce CD73 and CD39 expression (Dianzani et al., 1993; Raczkowski et al., 2018). In mice, we and others have demonstrated that CD73 is expressed on some T cell subsets, such as naïve and memory CD8+ T cells, and regulated during terminal effector differentiation (Heng et al., 2008; Flores-Santibanez et al., 2015). Despite this, the role of CD73 and CD73-generated adenosine in the differentiation of CD8+ T lymphocytes is currently unknown.

Here we report that CD73 restrains CD8+ T cell differentiation to Tc1 cells leading to reduced cytokine and granzyme B production. In agreement, CD73-deficient cells presented a higher commitment to the effector program with an increased glucose and oxygen consumption rate, indicating that this ectonucleotidase reduces the metabolic fitness in CD8+ T cells. In agreement, when adoptively transferred, antigen-specific CD73-deficient CD8+ T cells were more efficient in reducing the tumor burden in B16.OVA melanoma-bearing mice and presented a lower expression of exhaustion markers than wild type (WT) cells. All these data suggest an immunosuppressive autocrine effect of CD73-mediated adenosine production in restraining effector CD8+ T cell fitness and function.

## Materials and Methods

### Mice

CD73KO (B6.129S1-Nt5etm1Lft/J), C57BL/6 (CD45.2+), CD45.1+ (B6.SJL-Ptprca Pepcb/BoyJ), and OT-I mice were purchased from Jackson Laboratory. OT-I/CD73KO mice were obtained by backcrossing the F1(OT-I x CD73KO) with OT-I mice and testing for Vα2Vβ5 transgenic TCR and CD73 expression by FACS. All mice were kept in the animal facility at Fundacion Ciencia & Vida. Animal work was carried out under institutional regulations of Fundacion Ciencia & Vida and Facultad de Ciencias, Universidad de Chile, and was approved by the local ethics review committees.

### *In vitro* T Cell Differentiation

Naive CD8+ T cells were purified from spleens and lymph nodes of WT or CD73KO mice. Briefly, spleens were perfused with RPMI 1640 supplemented with 10% FBS, and lymph nodes were mechanically disgregated with scissors. The cell suspension was filtered through a metal mesh and CD8+ T cells were enriched by negative selection using MACS magnetic beads (Miltenyi Biotec) following the manufacturer’s instructions. Following enrichment of CD8+ T cells, naive CD8+ T cells (CD8+/CD44low/CD62Lhigh/CD25-) were obtained by cell sorting using a FACS Aria III cell sorter (Biosciences). Naive CD8+ T cells were cultured in 96-well round-bottom microplates (10^5^ cells/well) and were activated with soluble α-CD3 (1 μg/ml; clone 145-2C11, Biolegend) and α-CD28 (1 μg/ml; clone 37.51, Biolegend) for 3 days in the presence of 10 ng/ml of recombinant mouse IL-2 (eBioscience) to generate Tc1 cells.

### *In vivo* T Cell Differentiation

Naive CD8+ T cells were purified as described above from OT-I or OT-I/CD73KO mice. These cells were intravenously injected (i.v.) into CD45.1+ mice and 24 h later, the recipient mice received an intraperitoneal (i.p.) injection of OVA protein (500 μg, Sigma-Aldrich) plus LPS (25 μg, Sigma-Aldrich). On days 4, 7, 12, and 28 mice were euthanized and blood was drawn through cardiac puncture. Red blood cells were lysed using a RBC Lysis Buffer (eBioscience) for 5 min in ice. The cell suspension was then centrifuged, stained, and resuspended in PBS+ 2% FBS for FACS analysis.

### Flow Cytometry

Cellular suspensions were incubated with antibodies against CD16/CD32 (Fc block, Biolegend) for 15 min at 4°C in the dark to block Fc receptors. Subsequently, the cells were incubated for 30 min at 4°C in the dark with a mixture of antibodies conjugated with different fluorochromes in the presence of a viability dye (Fixable Viability Dye eFluor 780, eBiosciencies) to discard dead cells. The cells were then centrifuged at 600 × *g* for 7 min at 4°C and resuspended in PBS + 2% FBS for FACS analysis.

To assess cytokine production by *in vitro*-generated Tc1 cells, the cells were harvested at day 3 and restimulated with 0.25 μM PMA (Sigma-Aldrich) and 1 μg/ml ionomycin (Sigma-Aldrich) in the presence of GolgiPlug (BD Biosciences) for 4 h. Cells were stained with antibodies against cell surface markers and then resuspended in a fixation/permeabilization solution (Cytofix/Cytoperm; BD Pharmingen). Following fixation and permeabilization, the cells were incubated with antibodies against IFN-γ, IL-2, and TNF-α for 30 min at 4°C. The cells were then washed with permeabilization buffer and resuspended in PBS + 2% FBS for FACS analysis.

For intracellular granzyme B staining, following cell-surface staining, the cells were resuspended in a fixation/permeabilization solution (Foxp3 transcription factor fixation and permeabilization buffer, eBioscience) for 45 min at 4°C in the dark. Cells were washed with a permeabilization buffer (Foxp3 transcription factor buffer, eBioscience) and centrifuged at 700 × *g* for 7 min at 4°C. The cells were then incubated with anti-granzyme B antibody diluted in the same buffer for 30 min at 4°C in the dark. After intracellular staining, the cells were washed with the permeabilization buffer, centrifuged at 700 × *g* for 7 min at 4°C and resuspended in PBS+ 2% FBS to be analyzed by FACS. The cells were analyzed in a FACSCanto II flow cytometer (BD Bioscience).

For A2AR staining, the cells were harvested and resuspended in a fixation/permeabilization solution (Cytofix/Cytoperm; BD Pharmingen). After fixation and permeabilization, cells were incubated with anti-A2AR antibody (clone 7F6-G5-A2) from Novus Biologicals for 30 min at 4°C. Cells were then washed with permeabilization buffer (PermWash Buffer) and resuspended in PBS + 2% FBS for FACS analysis.

### Glucose Consumption by FACS

Tc1 cells were resuspended in glucose-free RPMI medium at a concentration of 0.5 × 10^6^ cells/mL and incubated with the fluorescent glucose analog 2-NBDG (100 μM, ThermoFisher) for 20 min at 37°C and 5% CO_2_ in the dark. After this incubation, the cells were washed twice with PBS and centrifuged at 600 × *g* for 7 min at 4°C. Finally, the pellet was resuspended in PBS + 2% FBS for FACS analysis.

### OCR and ECAR

Oxygen consumption rates (OCRs) and extracellular acidification rates (ECARs) from *in vitro*-generated Tc1 cells (0.5 × 10^6^ cells/well) were measured in non-buffered DMEM without phenol red (2 mM L-glutamine and 1 mM sodium pyruvate) under basal conditions and in response to 10 μM glucose, 1 μM oligomycin, 0.5 fluoro-carbonyl cyanide phenylhydrazone and 1 μM rotenone + 1 μM antimycin A + and 50 μM 2-DG (Sigma). The cells were analyzed with the XF24-3 Extracellular Flux Analyzer (Seahorse Bioscience).

### Adenosine Production

The production of adenosine was measured directly in the supernatants obtained during CD8+ T cell activation. For this, naive CD8+ T cells were cultured in 96-well plates at 10^5^ cells/well in the presence of soluble α-CD3 (1 μg/mL) and α -CD28 antibodies (1 μg/mL) and IL-2 (10 ng/mL). The culture medium was harvested on days 2 and 4 of activation, then centrifuged at 1000 × *g* for 10 min to discard cells and stored at –20°C for further analysis.

To evaluate adenosine production by Tc1 cells in the presence of AMP, Tc1 cells were differentiated for 3 days, harvested and diluted in Hanks’ balanced salt solution (HBSS). Cells were then incubated in 96-well flat-bottom plates at 0.5 × 10^5^ cells/well with AMP (10 μM, Sigma-Aldrich) in the presence or absence of the CD73 inhibitor APCP [Adenosine 5′-(*a,b*-methylene) diphosphate] (50 μM, Sigma-Aldrich). After 1 h, the cells were harvested, placed on ice for 15 min, and then centrifuged at 1000 × *g* for 10 min. Supernatants were collected and stored at –20°C until further analysis. Adenosine production was measured using the Adenosine Assay Kit (Cell Biolabs Inc.) following the manufacturer’s instructions.

### Real-Time PCR

Total RNA was extracted from *in vitro* differentiated CD73KO and wild type Tc1 cells. Naive CD8+ T cells were isolated by cell sorting (CD8+/CD44low/CD62Lhigh/CD25-) before RNA extraction. Total RNA was obtained using EZNA Total RNA Kit I (Ω Bio-Tek). RNA (1 μg) was reverse-transcribed using M-MLV reverse transcriptase (Promega). The PCR reaction was performed using Brilliant II SYBR Green QPCR Master Mix (Agilent Technologies) in a Stratagene Mx3000P real-time PCR machine. Fold change of gene expression was determined using the 2^–ΔΔ*Ct*^ method, which compares the expression level of each sample with WT naive CD8+ T cells. The following primers were used:

prdm1 forward 5′-GACGGGGGTACTTCTGTTCA-3′, reverse 5′-GGCATTCTTGGGAACTGTGT-3′
tbx21 forward 5′-AGCAAGGACGGCGAATGTT-3′, reverse 5′-GGGTGGACATATAAGCGGTTC-3′
hk-II forward 5′-TGATCGCCTGCTTATTCACGG-3′, reverse 5′-AACCGCCTAGAAATCTCCAGA-3′
cpt-1a forward 5′-CTCCGCCTGAGCCATGAAG-3′, reverse 5′-CACCAGTGATGATGCCATTCT-3′
hprt forward 5′-CTCCTCAGACCGCTTTTTGC-3′, reverse 5′-TAACCTGGTTCATCATCGCTAATC-3′.

### Tumor Growth

B16.OVA cells were kindly provided by Dr. Randolph Noelle (Dartmouth Medical School, Hanover, NH). B16.OVA cells (0.5 × 10^6^ cells) were injected into the intradermal layer of the right flank of mice. When tumors became visible at day 7, mice were injected i.v. with 10^6^ naive CD8 + T cells from OT-I or OT-I/CD73KO mice. The tumor size was measured every day. Two perpendicular measurements were made with a caliper, and the tumor area was calculated as the product of both measurements.

### Isolation of Tumor-Infiltrating Cells

Tumors were dissected and disaggregated mechanically. Minced tissues were resuspended in 5 ml Hanks’ balanced salt solution + 5% FBS and digested in the presence of 1 mg/ml collagenase D (Roche, Mannheim, Germany) and 25 mg/ml DNase I (Roche) for 30 min at 37°C with constant agitation. The cell suspension was filtered with a 70-μm cell strainer (BD Falcon, Franklin Lakes, NJ, United States). Red blood cells were lysed using a RBC lysing solution (Biolegend), centrifuged at 600 × *g* for 7 min, and stained for FACS analysis.

### Statistical Analysis

Data are presented as mean ± SEM. Differences between two groups were determined using the two-tailed Mann–Whitney test. Differences between more than two groups were determined using one-way ANOVA with Kruskal–Wallis Test. A two-way ANOVA with Bonferroni post-test was used to compare tumor growth. Statistical analysis and graphs were obtained with GraphPad PRISM (GraphPad Software Inc).

## Results

### CD73, CD39, and A2AR Are Upregulated *in vitro* During T Cell Activation

**FIGURE 1:**
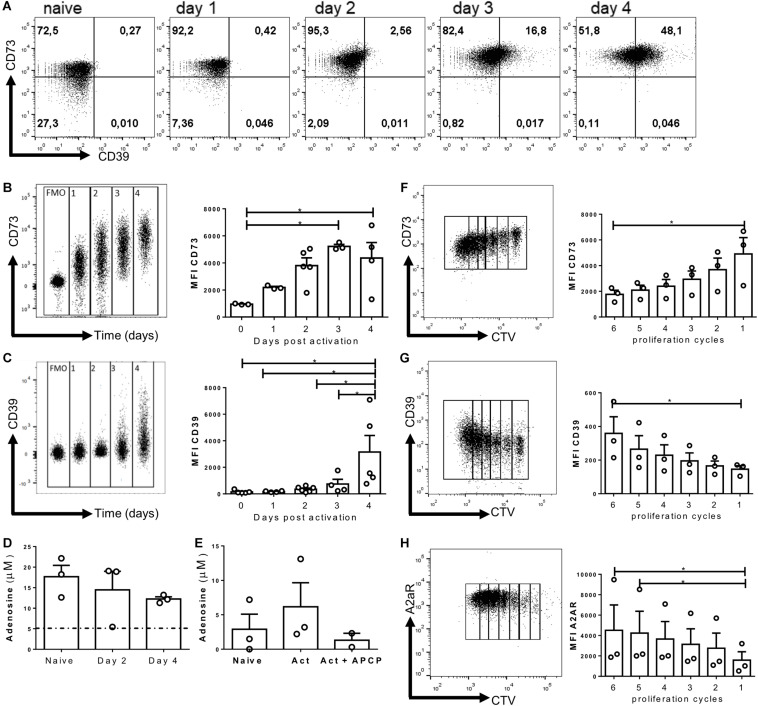
Purinergic components are upregulated during *in vitro* activation. Naive CD8+ T cells from WT mice were isolated and activated with soluble α-CD3/CD28 antibodies for 4 days *in vitro* in the presence of IL-2 (10 ng/ml) for the generation of Tc1 lymphocytes. **(A)** Representative dot plots of CD39 and CD73 expression by *in vitro*-generated Tc1 cells at different time points. **(B,C)** Representative graph showing the concatenation and MFI of CD73 **(B)** and CD39 **(C)** on T cells at different days following activation. **(D)** Adenosine concentration obtained from naïve cells and *in vitro* Tc1 cultures at days 2 and 4 following activation. **(E)** Adenosine production by Tc1 cells cultured for 1 h in the presence of AMP (10 μM) and APCP (50 μM). **(F–H)** Representative dot plots and MFI graphs showing CD73 **(F)**, CD39 **(G)**, and A2AR **(H)** expression as a function of Tc1 proliferation cycles (at day 3) as assessed by cell trace violet dilution by FACS. Kruskal–Wallis Test, *p < 0.05, *n* = 3, Data is presented as the mean ± SEM.

To understand the role of CD73 ectonucleotidase and adenosine during CD8+ effector cell differentiation, we first evaluated the kinetics of expression of CD73 and CD39 on *in vitro*-activated CD8+ T lymphocytes. As shown in Figure 1A, naïve CD8+ T cells express high levels of the CD73 ectonucleotidase but do not express CD39. Upon activation, CD73 is further upregulated, reaching a peak of expression by day 3 (Figure 1B). Interestingly, CD39 also becomes upregulated 3 days following activation (Figures 1A,C), suggesting that activated T cells express all the enzymes needed to hydrolyze ATP to adenosine. Next, we sought to evaluate whether activated CD8+ T cells produce adenosine upon activation. As shown in Figure 1D, CD8+ T cells produce adenosine, and the concentration of this nucleoside is maintained following *in vitro* activation. However, when the cells were pre-incubated with AMP (the substrate of CD73), activated T cells produced higher levels of adenosine that naïve T cells and this was reversed by the addition of the CD73 enzymatic activity inhibitor APCP (Figure 1E). In conclusion, these results suggest that CD8+ T cells express CD39 and CD73 enzymes and produce adenosine upon *in vitro* activation.

Next, we analyzed the expression of purinergic signaling components in dividing cells 3 days post-activation. To this end, naive T cells were labeled with Cell Trace Violet, and at day three post-activation, we evaluated the expression of CD73, CD39, and A2AR at each round of proliferation. We observed that the expression of CD73 is down-regulated as a function of the proliferation cycles (Figure 1F), whereas CD39 and A2AR expression is upregulated as cells proliferate (Figures 1G,H). Despite the evident reduction in CD73 expression between the different proliferation rounds on day 3, we consistently observed an increase in the mean fluorescence intensity of CD73 in CD8+ T cells between days 1 and 4 after *in vitro* activation (Figures 1A,B). These results indicate that the expression of purinergic signaling components is positively regulated on CD8+ T cells under conditions that induce their differentiation to effector cells.

### CD73 and CD39 Are Upregulated During an *in vivo* Immune Response

Our results presented so far indicate that purinergic components are upregulated on CD8+ T cells during *in vitro* activation. In light of these results, we decided to evaluate the expression of CD73 and CD39 ectonucleotidases during an *in vivo* immune response at the effector (12 days) and memory phase (28 days). For this, we adoptively transferred naive CD45.2+ OT-I lymphocytes into CD45.1+ recipient mice, and 24 h later, all mice received an i.p. injection of OVA protein.

**FIGURE 2:**
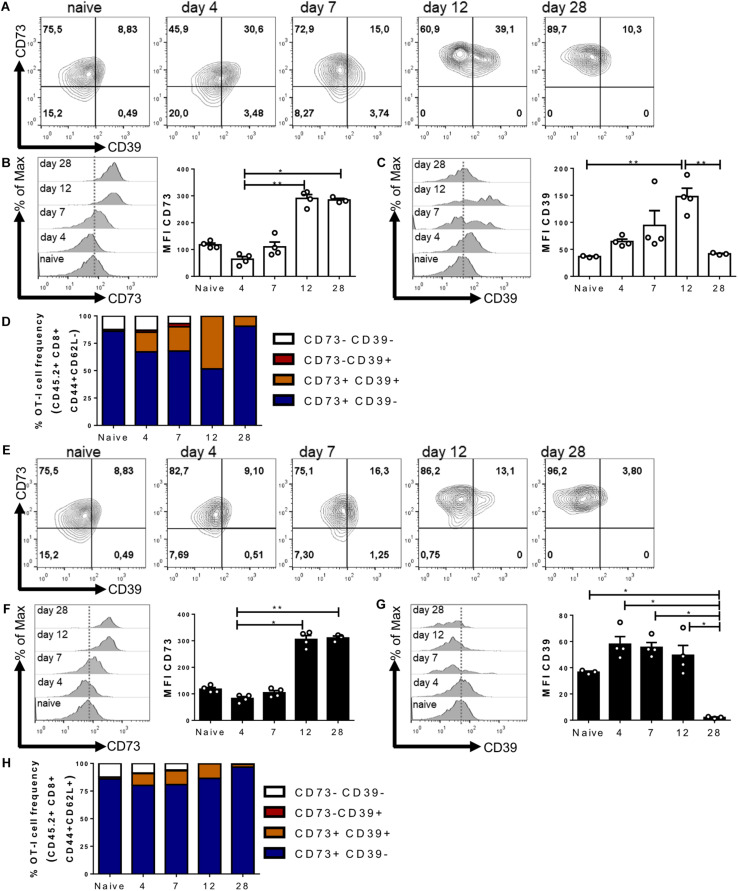
CD39 and CD73 ectonucleotidases are upregulated during an *in vivo* immune response. Naive OT-I cells (10^6^ cells) were injected into CD45.1+ recipient mice. 24 h later, the recipient mice were immunized i.p with OVA protein (500 μg) + LPS (25 μg). Transferred cells were analyzed in blood samples on days 4, 7, 12, 21, and 28 after the immunization. **(A–D)** CD39 and CD73 expression was analyzed in the effector memory precursors (CD45.2+/CD8+/CD44+/CD62L–) or central memory precursors (CD45.2+/CD8+/CD44+/CD62L+) **(E–H)** at different time points. *n* = 4, Kruskal–Wallis Test, *p < 0.05; **p < 0.01.

When analyzing transferred antigen-specific CD8+ T cells (Figures 2A–D), we first assessed the CD62L– population that has been identified as having a greater commitment to effector differentiation (Gattinoni et al., 2012). In agreement with our *in vitro* assays, we observed a change in the expression levels of both CD73 and CD39 as a function of T cell activation (Figure 2A). We observed a transient down-regulation on CD73 expression by day four, followed by a gradual increase in its expression during the memory phase (Figure 2B). On the other hand, the transferred cells acquired CD39 expression during the effector phase (peaking at day 12), followed by a gradual reduction in expression in the memory phase (Figure 2C). Interestingly, the frequency of CD8+ T cells expressing both CD39 and CD73 ectonucleotidases peaked during the effector phase on day 12 (Figure 2D).

Next, we analyzed the population of cells that maintains the expression of CD62L and thus is less committed to an effector differentiation (Figures 2E–H) (Sallusto et al., 2004). In these cells, we also observed a transient reduction in CD73 expression on day four and a gradual increase at later time points during the memory phase (Figures 2E,F). On the other hand, CD39 expression is only expressed by a small fraction of these cells during the effector phase on day 12 (Figures 2E,G,H). The frequency of CD8+ T cells that express only CD73 is maintained throughout the immune response (Figure 2H). These results are in agreement with our *in vitro* experiments and indicate that the expression of ectonucleotidases is positively regulated during the activation phase of the immune response, with CD39 and CD73 being concomitantly expressed mainly by effector cells.

### CD73 Restrains the Effector Program of CD8+ T Cells

**FIGURE 3:**
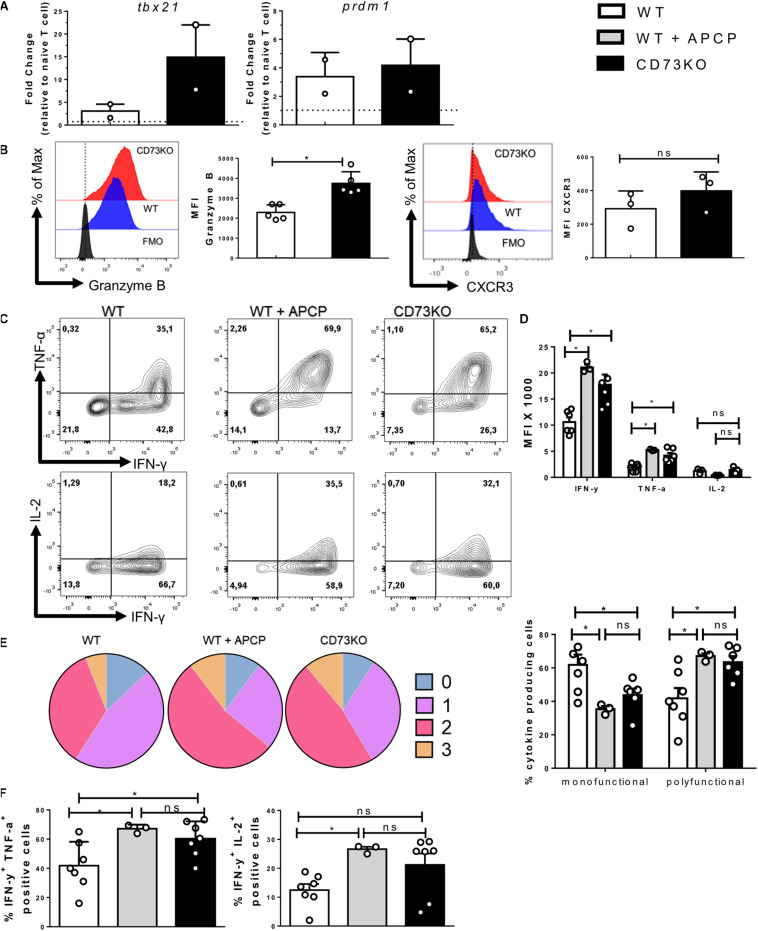
CD73 limits the acquisition of the Tc1 effector program. Naive CD8+ T cells from WT mice were isolated and activated with soluble α-CD3/CD28 antibodies for 3 days *in vitro* in the presence of IL-2 (10 ng/ml) for the generation of Tc1 lymphocytes. Transcription factors, cytotoxic proteins, and cytokine production by Tc1 cells were evaluated by real-time PCR and FACS. **(A)** Expression of *prdm1* and *tbx21* was measured by real-time PCR (*n* = 2). **(B)** Left, Representative histogram showing granzyme B production (*n* = 5). Right, histogram depicting CXCR3 expression (*n* = 3). **(C)** Representative dot plots showing TNF-α, IFN-γ, and IL-2 production by CD73KO and WT CD8+ T cells in the presence and absence of CD73 enzymatic activity inhibitor APCP (50 μM) (*n* = 3–7). **(D)** Bar graph showing the MFI for IFN-γ, TNF-α, and IL-2. **(E)** Left, graph showing the frequency of Tc1 cells that produce 0, 1, 2, and 3 cytokines. Right, frequencies of Tc1 cells producing up to one cytokine (mono-functional) or more than two cytokines (poly-functional). **(F)** Bar graphs showing the percentage of cells that produce IFN-γ and TNF-α or IFN-γ and IL-2. Two-tailed Mann–Whitney test and Kruskal–Wallis Test, *p < 0.05. Data is presented as the mean ± SEM.

Our results confirm the presence of a population of effector cells that express both ectonucleotidases, which enables adenosine production. Next, we analyzed whether this could represent a physiological mechanism to avoid an excessive effector response on CD8+ T cells. Therefore we analyzed some hallmarks of the effector program of Tc1 cells such as transcription factors, granzyme B, and cytokines produced by *in vitro*-differentiated WT and CD73KO CD8+ T cells. As shown in Figure 3A, our results demonstrate that the expression of transcription factors related to the acquisition of the effector program, such as *tbx21* (T-bet), *prdm1* (Blimp-1) are upregulated in both WT and CD73KO CD8+ T cells, but the expression of tbx21 is higher in CD73KO compared to WT CD8+ T cells.

Accordingly, CD73KO CD8+ T cells presented higher expression of granzyme B compared to WT cells (Figure 3B), suggesting that CD73 reduces the cytotoxic potential of CD8+ T lymphocytes. On the other hand, we evaluated the expression of CXCR3, a chemokine receptor that is required for rapid migration to inflamed tissues (Maurice et al., 2019). Our results indicate that the absence of CD73 does not affect the expression of this receptor (Figure 3B).

Finally, we observed a higher production of pro-inflammatory cytokines such as IFN-γ and TNF-α in CD73-deficient lymphocytes compared to WT CD8+ T cells (Figures 3C,D). The same result was obtained when CD73 enzymatic activity was ablated using the CD73 specific inhibitor APCP. When assessing CD8+ T cells’ ability to produce multiple cytokines simultaneously, our results indicate that CD73KO CD8+ T cells and APCP treated WT cells show an expanded polyfunctionality when compared to WT lymphocytes (Figures 3E,F). These results suggest that CD73 expression and adenosine production reduces the polyfunctionality and the cytotoxic potential of CD8+ T cells.

### CD73 Restricts the Mitochondrial Capacity in Activated CD8+ T Cells

**FIGURE 4:**
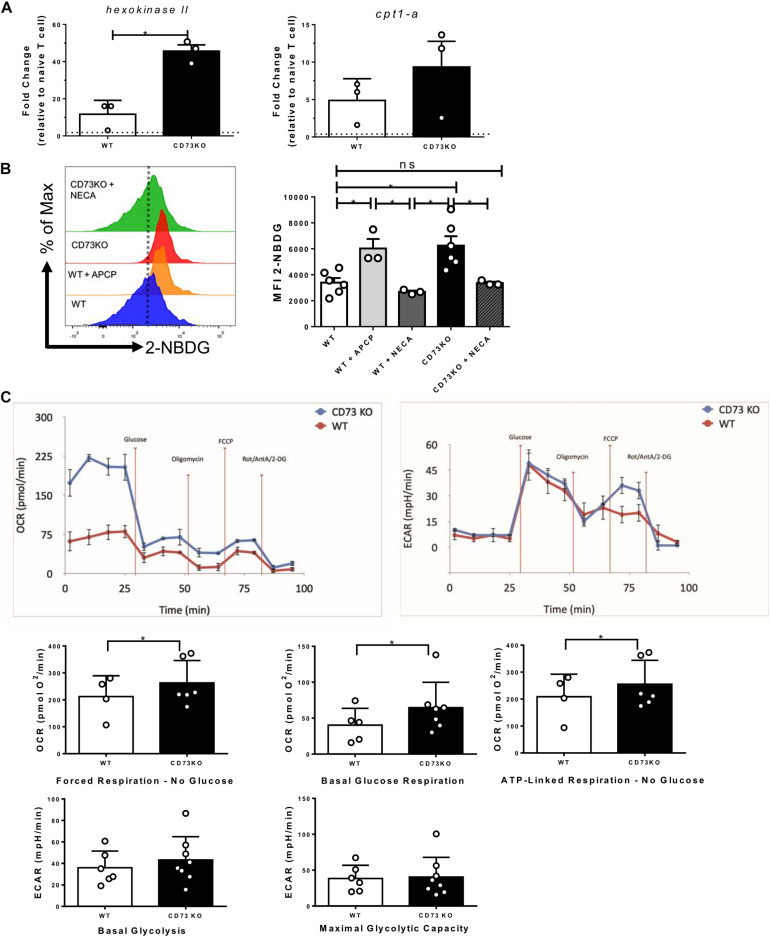
CD73 restricts the mitochondrial capacity of Tc1 cells. Naive CD8+ T cells from WT mice were isolated and activated with soluble α-CD3/CD28 antibodies for 3 days *in vitro* in the presence of IL-2 (10 ng/ml) for the generation of Tc1 lymphocytes. **(A)** Expression of *hexokinase II* and *cpt-1* mRNAs was analyzed by real-time PCR in CD73 and WT Tc1 cells (*n* = 3). **(B)** Glucose uptake of WT and CD73KO Tc1 cells treated with APCP (50 μM) or NECA (10 μM) was measured by FACS using the fluorescent glucose analog 2-NBDG (*n* = 3–6). **(C)** Left panel: Oxygen consumption rates (OCRs) and Right panel: extracellular acidification rates (ECARs) from CD73KO and WT Tc1 cells were measured under no glucose (forced mitochondrial respiration), basal glucose respiration and ATP-linked respiration for OCR and basal glycolysis and oligomycin stimulated glycolysis (maximal glycolytic capacity) for ECAR (*n* = 4;6 Tc1 WT and 8 CD73KO Tc1) Kruskal–Wallis Test, Paired *T*-test and Two-tailed Mann–Whitney test, *p < 0.05. Data is presented as the mean ± SEM.

Cytotoxic lymphocytes present a glycolytic metabolism that allows the generation of precursor metabolites for biosynthesis and energy generation processes (Vander Heiden et al., 2009; MacIver et al., 2013). Due to our previous results showing that CD73 reduces the effector program of Tc1 cells, we asked whether this is related to their bioenergetic capacity. As a first approach, we evaluated the mRNA expression of two critical metabolic pathway regulators, *hexokinase II* for the glycolytic pathway (Tanner et al., 2018) and *cpt1-a* for the FAO pathway (van der Windt and Pearce, 2012) in WT and CD73 deficient CD8+ T cells following activation. The results indicate that the absence of CD73 promotes *hexokinase II* and *cpt-1* mRNA expression (Figure 4A).

Given these results, we used 2-NBDG, a fluorescent glucose analog to evaluate glucose uptake. As seen in Figure 4B, the absence of CD73 in CD8+ T lymphocytes significantly improves the ability to uptake glucose compared to WT lymphocytes. Similar results were obtained when CD73 enzymatic activity was inhibited with APCP (Figure 4B). Moreover, when CD73KO CD8+ T cells are incubated with the A2A receptor agonist NECA, the ability of the cells to uptake glucose is significantly reduced to a level similar to WT cells (Figure 4B).

To elucidate which metabolic pathway, glycolysis or OXPHOS, is affected in CD73KO cells, we run a seahorse assay to determine the OCR and ECAR with a media containing glutamine and pyruvate as respiratory substrates but no glucose. With this media, cells are forced to use mitochondria and then OXPHOS to drive ATP synthesis (forced respiration). Under these conditions, our results showed that CD73KO cells have a much higher OCR than WT cells, meaning a higher bioenergetic mitochondrial capacity. CD73KO cells also exhibited a significantly higher mitochondrial respiration in the presence of glucose than WT cells (basal glucose respiration). In addition, ATP-linked respiration in the absence of glucose was also higher in CD73KO cells. No significant differences were found in oligomycin insensitive respiration nor FCCP induced respiration. Of note, forced mitochondrial respiration in the absence of glucose represents the maximal respiratory capacity in these cells.

On the other hand, no significant differences were found regarding the basal glycolysis (after glucose injection) and maximal glycolytic capacity between CD73KO and WT cells (Figure 4C). These results suggest that the increased glucose uptake shown by CD73KO cells is used for mitochondrial metabolism.

### CD73 Reduces the Antitumoral Activity of Tumor-Infiltrating CD8+ T Cells

**FIGURE 5:**
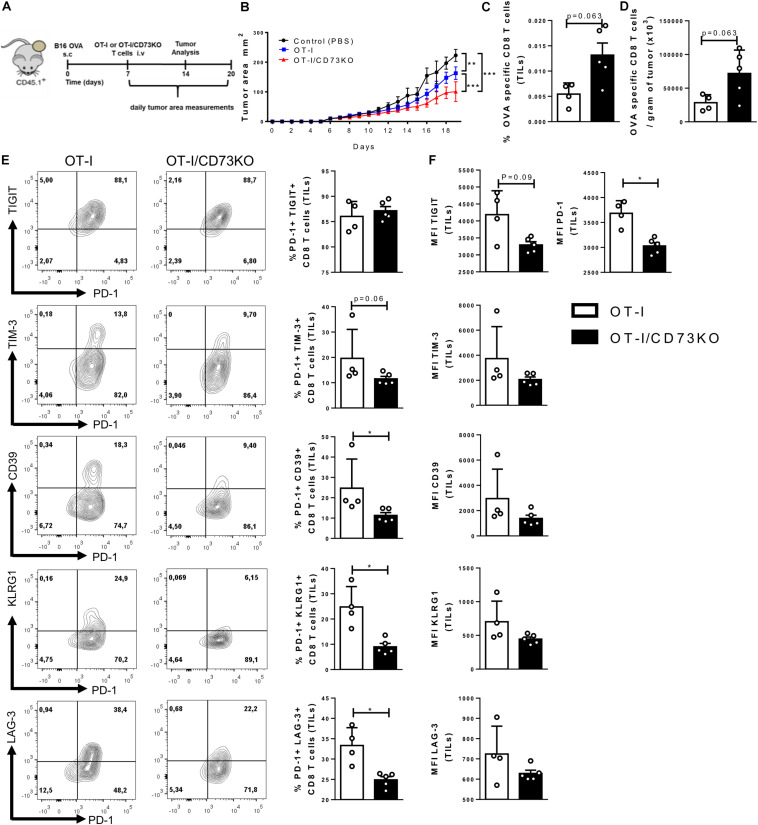
CD73-deficient CD8+ T cells are more effective in controlling tumor growth than WT cells. **(A)** Diagram showing the experimental setup: B16.OVA melanoma cells were intradermally injected in the back right flank of CD45.1+ mice. Seven days after the tumor challenge, OT-I or OT-I/CD73KO CD8+ naïve T cells (1 × 10^6^ cells) were adoptively transferred. Tumor area and tumor-infiltrating T cells were analyzed. **(B)** Tumor growth curves of CD45.1+ mice that were injected with OT-I or OT-I/CD73KO CD8+ naïve T cells or control (PBS) (*n* = 5). Tumor area was calculated by measuring and multiplying perpendicular axes, the results were plotted as the mean ± SEM for each day. **(C)** The absolute number of OVA-specific CD8+ T cells per gram of tumor (*n* = 5). **(D)** Frequency of OVA-specific CD8+ T cells in the tumor (TILs) (*n* = 5), **(E)** Dot plots, and bar graphs depicting the expression and frequency of positive cells within the adoptively transferred cells (CD45.2+/CD8+ gate) for the different inhibitory receptors. **(F)** MFI of inhibitory receptors in the adoptively transferred cells (CD45.2+/CD8+ gate). Data is presented as the mean ± SEM. Significance was determined using two-way repeated measured ANOVA with Bonferroni’s correction *post hoc* at day 19 **(B)**, and unpaired two-tailed Mann–Whitney test **(C–E)**. *p < 0.05, **p < 0.01, ***p < 0.001, ****p < 0.0001 (**B**, Two-Ways ANOVA), (**C–E**, Two-tailed Mann–Whitney Test).

Since our results suggest that CD73 expressed by activated CD8+ T cells has a role in reducing the effector phenotype of these cells we compared the anti-tumoral activity of WT and CD73KO CD8+ T cells. To this end, we challenged CD45.1+ mice with an intradermal injection of B16.OVA cells. When tumors became visible, we transferred 1 × 10^6^ WT-OT-I or CD73-deficient OT-I naïve T cells (OT-I/CD73KO) (Figure 5A). Our results confirm that tumor growth was significantly reduced in mice that were adoptively transferred with either OT-I or OT-I/CD73KO CD8+ T cells compared with control mice (PBS); however, the transfer of OT-I/CD73KO CD8+ T cells had a more dramatic effect on tumor growth than the transfer of OT-I CD8+ T cells (Figure 5B). Moreover, we observed an increase in the percentage and numbers of OVA-specific CD8+ T cells per gram of tumor in mice that were transferred with OT-I/CD73KO CD8+ T cells compared with mice transferred with OT-I cells (Figures 5C,D).

Given these results, we asked whether the ability of CD8+ T cells to produce adenosine constitutes a regulatory signal for the expression of inhibitory checkpoint receptors such as PD-1, TIGIT, TIM-3, CD39, KLRG1, and LAG-3. In agreement, the co-expression of inhibitory receptors was higher in tumor-infiltrating OT-I lymphocytes compared to OT-I/CD73KO cells (Figure 5E). Although there are no differences in the relative percentages of PD-1 and TIGIT co-expression, OT-I lymphocytes have a higher degree of TIM-3, LAG3, KLRG1, and CD39 expression compared to CD73KO (Figure 5E). Additionally, OT-I lymphocytes have a higher mean PD-1 fluorescence intensity than OT-I/CD73KO lymphocytes (Figure 5F). These results indicate that CD73 expression by CD8+ T lymphocytes reduces the anti-tumoral capacity of CD8+ cells and increases the degree of CD8+ T cell exhaustion.

## Discussion

CD73 has been proposed as a novel immune checkpoint, and thus several strategies to block adenosine production by this enzyme have been suggested (Beavis et al., 2012; Vigano et al., 2019). However, the impact of these strategies on CD8+ T cell effector function has not been previously addressed. Here we report on the role of CD73 ectonucleotidase on CD8+ T cell differentiation and its effector program. Our results provide evidence that adoptive transfer of CD73-deficient CD8+ T cells into tumor bearing mice show a higher frequency and number of tumor-infiltrating lymphocytes and are more effective in controlling tumor growth than WT cells. These results are supported by our data showing that during *in vitro* differentiation, CD8+ T cells upregulate the expression of CD73 and CD39 ectonucleotidases, A2A receptor, and produce adenosine. Accordingly, CD73 deficiency promoted effector functions by increasing cytokine and granzyme B production, metabolic fitness, and the anti-tumoral activity of CD8+ T cells. All these results suggest that CD73 ectonucleotidase restrains the effector program of CD8+ T cells through adenosine production.

Different reports have described the expression of CD39 and CD73 ectonucleotidases in an inflammatory context (Antonioli et al., 2013; Allard et al., 2017), but most of these reports focus on the presence of these molecules in tumor cells or regulatory cells, overlooking other populations such as CD8+ T cells. Our results from *in vitro* and *in vivo* experiments show that naive CD8+ cells express CD73, and this enzyme is upregulated following T cell activation during the effector and memory phases. On the other hand, CD39 expression becomes relevant during the effector phase, peaking at day 12. In memory precursor cells (CD44hi/CD62L+), CD73 expression increases during the effector and memory phases, but CD39 expression in this cell subset always remains very low. These results indicate that CD39 is expressed in cells with a higher degree of commitment to the effector program and are therefore more prone to apoptosis (Fang et al., 2016) or cellular exhaustion (Gupta et al., 2015). Taken together, these data suggest that the concomitant expression of CD39 and CD73 during the effector phase enables CD8+ T cells to produce adenosine from extracellular ATP. Our results do not discard the possibility that at early phases during activation, CD8+ T cells, and even naïve T cells may produce adenosine from AMP as they express CD73.

The A2AR is the primary receptor mediating adenosine signaling in T cells, and our results confirm that CD8+ T cells rapidly upregulate this receptor following activation. Stimulation of A2AR increases cAMP production and activation of protein kinase A, signals that have been described to weaken TCR-mediated signaling (Vang et al., 2001; Linnemann et al., 2009; Linden and Cekic, 2012; Rodriguez et al., 2013; Newick et al., 2016). Our results with APCP, a specific inhibitor of CD73 enzymatic activity, recapitulate some of the characteristics we observed in CD73 deficient CD8+ T cells, including the increase in cytokine production, and glucose uptake, suggesting that at least these features may depend on CD73-mediated adenosine production and A2AR signaling. In agreement with these results, the disruption of A2AR signaling improved the production of type 1 cytokines such as TNF-α and IFN-γ by CD4+ and CD8+ T cells (Munoz et al., 1990; Erdmann et al., 2005; Raskovalova et al., 2007; Mastelic-Gavillet et al., 2019). Additional experiments designed to analyze other effector functions on WT CD8+ T cells incubated with A2AR antagonists or CD73KO CD8+ T cells cultured in the presence of adenosine receptor agonists could further support our hypothesis that CD73 may be promoting autocrine adenosine signaling in CD8+ T cells.

The ability to uptake glucose has been related to IFN-γ production, as glycolysis has been shown to regulate its expression by epigenetic and post-translational mechanisms (Chang et al., 2013; Peng et al., 2016). Similarly, it has been described that an increase in aerobic glycolysis promotes the production of other effector molecules, such as granzyme B (Cham and Gajewski, 2005; Cham et al., 2008). Interestingly, it has been suggested that aerobic glycolysis promotes the accumulation of cytoplasmic calcium, leading to the maintenance of the NFAT transcription factor needed to fulfill the effector function of T cells (Ho et al., 2015; Klein-Hessling et al., 2017; Gemta et al., 2019). All together, our results suggest that CD73-mediated adenosine production by CD8+ T cells may restrain their effector function by restricting the ability of CD8+ T cells to uptake glucose.

Here we show that CD73 deficient Tc1 cells express higher levels of the *hexokinase II* mRNA, a “metabolic checkpoint” and a key player in the glycolytic flow, catalyzing the first step of glycolysis (Marin-Hernandez et al., 2006; Tanner et al., 2018). However, our results showed that the loss of CD73 has no effect on the glycolytic capacity of Tc1 cells, but rather on mitochondrial metabolism. It has been described that the subcellular location of hexokinase near the outer membrane of mitochondria facilitates the coupling between glycolysis and OXPHOS (Fiek et al., 1982; Linden et al., 1982; Arora and Pedersen, 1988; Roberts and Miyamoto, 2015) which may explain why the increased *hexokinase II* expression in CD73KO cells is associated with higher OCR levels. Although the expression of cpt1-a is associated with memory T lymphocytes (van der Windt et al., 2012), our results are consistent with reports describing an increase in its expression after activation in effector lymphocytes (Byersdorfer et al., 2013; Klein Geltink et al., 2017). Further, it has been associated with the fact that extracellular lipids play a key role in anabolic processes, and that a reduction in their availability reduces metabolic activity (O’Sullivan et al., 2014). However, further studies are needed to evaluate the degree of use of FAO through the use of cpt-1 inhibitors (such as etomoxir) and exogenously added fatty acids such as palmitate.

Recent evidence demonstrated that there is a metabolic competition between cancer and T cells for glucose uptake (Chang et al., 2015) where the increased glycolytic rate in tumors can inhibit the antitumor function of T cells by deprivation of glucose (Chang et al., 2015; Ho et al., 2015). Our data indicate that CD73 through adenosine production may control glucose uptake in CD8+ T cells, resulting in a reduced efficiency of T cells to control tumor burden. Moreover, when mice were adoptively transferred with CD73 deficient naïve OT-I cells, we observed a higher frequency and number of tumor-infiltrating lymphocytes (TILs) compared with mice transferred with OT-I cells. In addition, CD73 deficient cells presented a reduction in the expression of exhaustion markers such as PD1, CD39, TIM-3, and TIGIT, compared to WT cells, suggesting that CD73 expression may favor an exhausted phenotype on CD8+ T cells. In this line, it has been demonstrated that PD-1 signaling leads to a reduction in glycolysis, which may in turn promote the exhaustion of T cells (Bengsch et al., 2016). Furthermore, other studies have shown that mitochondrial function is needed to maintain optimal effector functions within the tumor microenvironment and that metabolic stress generated by nutrient deprivation leads to an impaired mitochondrial function (Scharping et al., 2016; Siska et al., 2017; Yu et al., 2020). In summary, our results suggest that CD73-mediated adenosine production alters glucose uptake by CD8+ T lymphocytes, which causes the cells to be less functional by reducing their energy generation capacity, which leads to exhaustion.

Bulk evidence from the literature demonstrates that CD73 expression in human CD8+ T cells is somehow comparable to murine CD8+ T cells. It has been described that human and murine CD8+ naïve T cells present the highest expression of CD73 among CD8+ T cells (Tóth et al., 2013; Raczkowski et al., 2018). Interestingly, Raczkowski et al. (2018) demonstrate that human CD8+ T cells experience a transient upregulation on CD73 expression following *in vitro* T cell activation (Tóth et al., 2013; Raczkowski et al., 2018), which is similar to what is observed in murine cells. Finally, several reports in humans and mice have shown that CD8+ T cells which are chronically stimulated or present in inflamed sites or tumors are mostly CD73–CD39+ cells which express exhaustion markers (Moncrieffe et al., 2010; Botta Gordon-Smith et al., 2015; Gourdin et al., 2018; Kong et al., 2019). In agreement, we have previously demonstrated that a subpopulation of murine CD8+ T cells presenting an effector memory phenotype and CD8+ T cells that infiltrate tumors express high levels of CD39 and lack CD73 expression (Flores-Santibanez et al., 2015). Thus, as for murine CD8+ T cells, CD73 expression in human CD8+ T cells is mostly ascribed to naïve cells and downregulated in exhausted cells.

## Conclusion

Our results provide compelling evidence with clinical significance since the use of blocking antibodies against CD73 could promote the effector cytotoxic capacity in CD8+ T cells leading to control tumor development. Different reports have described, in preclinical models, that CD73 blockade improves antitumor capacity (Stagg et al., 2010; Hay et al., 2016; Allard et al., 2017). In view of these results, interventions that target the generation of adenosine may not only prevent tumor-derived immunosuppression but also intrinsically enhance the metabolic fitness and cytotoxic activity of CD8 T+ lymphocytes.

## Data Availability Statement

The raw data supporting the conclusions of this article will be made available by the authors, without undue reservation.

## Ethics Statement

The animal study was reviewed and approved by Comité Institucional de Cuidado y Uso de Animales (CICUA) – Universidad de Chile.

## Author Contributions

PB and ER-Y performed the experiments, analyzed the data, and wrote the manuscript. MVR, BP-T, PF, LV, and VS performed the experiments and analyzed the data. AE, FS-O, AL, and CC analyzed the data and wrote the manuscript. DS, MB, and MR designed the study and wrote the manuscript. All the authors critically read the manuscript.

## Conflict of Interest

The authors declare that the research was conducted in the absence of any commercial or financial relationships that could be construed as a potential conflict of interest.
